# Rumen protozoa and viruses: The predators within and their functions—A mini-review*

**DOI:** 10.3168/jdsc.2023-0433

**Published:** 2024-02-01

**Authors:** Zhongtang Yu, Ming Yan, Sripoorna Somasundaram

**Affiliations:** Department of Animal Sciences, Center of Microbiome Science, The Ohio State University, Columbus, OH 43210

## Abstract

The rumen microbiome digests plant feedstuff that would be otherwise indigestible and provides most of the metabolizable energy and protein the host animals need. Until recently, research efforts have primarily been directed to bacteria and archaea, leaving the protozoa, fungi, and viruses much less understood. Protozoa contribute to feed digestion and fermentation, but as predators, they affect the microbiome and its function by regulating the abundance and activities of other rumen microbes both in a top-down (by directly killing the prey) and bottom-up (by affecting the metabolism of other microbes) manner. Rumen viruses (or phages, used interchangeably below) are diverse and abundant but the least understood. They are also predators (intracellular “predators”) because of their lytic lifecycle, although they can co-exist peacefully with their hosts and reprogram host metabolism, buttressing host ecological fitness. In doing so, rumen viruses also affect the rumen microbiome in both a top-down and a bottom-up manner. Here we review the recent advancement in understanding both types of predators, focusing on their potential impact on the rumen microbiome and functions.

## Graphical Abstract

**Figure:**
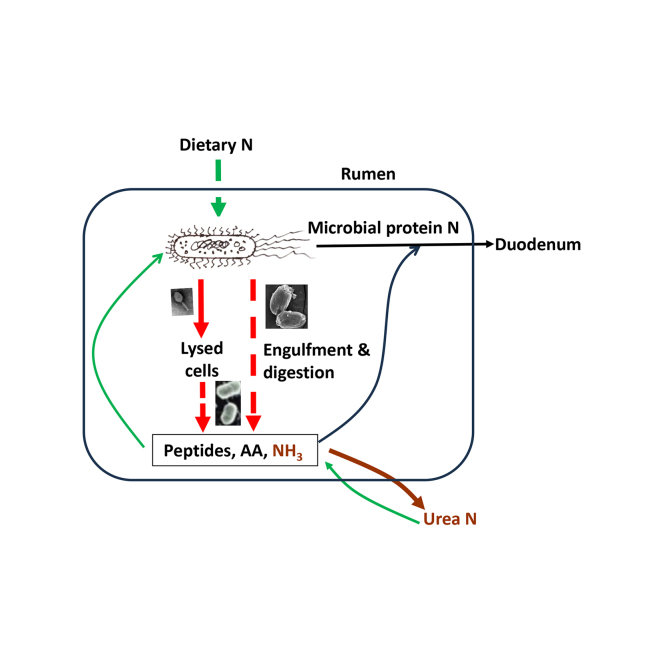

Dairy cows, goats, and sheep depend on the rumen microbiome for their livelihood and most of the precursors of milk production. This microbiome is taxonomically and functionally diverse, consisting of bacteria, archaea (primarily methanogens), protozoa (nearly exclusively ciliates), fungi, and viruses as different guilds performing feed digestion and fermentation. Each guild has its niche and plays a distinct, though often overlapping, role affecting rumen function and feed efficiency. Contemporary metataxonomics and metagenomics greatly expand the scope of diversity of the rumen microbiome, particularly rumen bacteria and archaea.

Protozoa are predators in the rumen, whereas viruses infect and lyse their host. Both form predator-prey relationships with other rumen microbes and can profoundly affect the rumen microbiome in both a top-down (by killing the prey) and a bottom-up (by affecting the metabolism of their host microbes) manner (Yan et al., 2023), exerting a far-reaching impact on rumen functions, especially fiber digestion, protein metabolism, and methane (CH_4_) emissions. However, both rumen protozoa and viruses are much less studied and understood. Recent studies employing omics technologies have shone new light on these 2 groups of rumen microbes. Here, summarizing the findings of recent omics studies on rumen protozoa, rumen virome, and the viromes in other ecosystems, we provide an elaborate narrative about these 2 groups of predators in the context of rumen functions.

Rumen protozoa exclusively inhabit the rumen and similar anaerobic habitats. Due to their large size, protozoa constitute up to 50% of the rumen microbial biomass (Andersen et al., 2023). Rumen protozoa have been morphologically categorized as entodiniomorphids and holotrichids, belonging to *Entodiniomorphida* and *Vestibuliferida*, respectively. Rumen protozoa are dominated by the genus *Entodinium*, although their abundance varies depending on host species and diet. In vitro and defaunation studies have revealed that rumen protozoa play important roles in feed digestion and fermentation (Newbold et al., 2015), but they are much less understood compared with their prokaryotic cohabitants. This is primarily attributed to the difficulty in obtaining maintainable axenic cultures for detailed biological characterization (Park et al., 2017). Thanks to recent genomic, transcriptomic, and proteomic studies, more direct and definitive evidence is emerging to define the biological characteristics of rumen protozoa and their impact on rumen functions.

Some rumen protozoa digest dietary fiber as evidenced by decreased fiber digestibility in defaunated animals (Newbold et al., 2015). Although the lack of axenic culture prevents definitive evidence, the recent discoveries of glycoside hydrolases (**GH**) such as cellulases and xylanases and their coding genes (Park et al., 2021; Li et al., 2022) and transcripts (Wang et al., 2019) attest to their ability to digest dietary fiber. Interestingly, *Diplodiniinae* and *Ophryoscolecinae* species possess as many GH genes as gut fungi, with ∼80% encoding GH acting on dietary fiber (Li et al., 2022). Comparative genomic analyses suggest that rumen protozoa acquired most of their GH-coding genes from bacteria through horizontal gene transfer (Park et al., 2021; Li et al., 2022). Interestingly, the verified activities of one cellulase and one xylanase of protozoa were 2 to 9 times higher than those of the inferred bacterial gene donors (Li et al., 2022). Additionally, protozoa enhance bacterial fiber digestion, probably by scavenging the free oxygen entering the rumen and thus helping maintain anaerobiosis, a requirement for strictly anaerobic fibrolytic bacteria. While we cannot directly measure oxygen consumption by rumen protozoa due to the lack of axenic cultures, transcriptomic (Wang et al., 2019) and proteomic data (Andersen et al., 2023) support the notion of oxygen consumption and detoxification of reactive oxygen. Ongoing omics studies will provide further insight into the capability and mechanism of fiber digestion by rumen protozoa.

Rumen protozoa can attenuate the sharp decrease of rumen pH in animals fed high-concentrate diets. The attenuation is likely attributable to their ability to uptake sugars (Nagaraja and Titgemeyer, 2007) and sequester them as glycogen (Denton et al., 2015), thereby ostensibly reducing the availability of readily fermentable substrates for bacterial fermentation. Indeed, *Entodinium caudatum* stores large glycogen granules (Park et al., 2017), despite being considered to store less glycogen than species *Dasytricha* and *Isotricha*. Conceivably, such moderation of pH drops reduces rumen acidosis. A recent genomic study revealed the genes involved in glycogen synthesis and utilization in *En. caudatum* (Park et al., 2021), while transcriptomics (Wang et al., 2019) and metaproteomics (Andersen et al., 2023) verified the expression of many of these genes. However, defaunation has little effect on rumen pH (Newbold et al., 2015). Diet is probably a major influential factor.

Through fermentation, rumen protozoa produce VFA, contributing to the major ME for ruminants. No conclusive evidence is available for the specific VFA produced by individual protozoal species due to the lack of axenic cultures. Data from studies using monocultures of *En. caudatum* and *D. ruminantium* suggest that the former produces acetate, butyrate, and propionate as the major VFA (Park et al., 2019a), whereas the latter primarily produces lactate, butyrate, and acetate (Ellis et al., 1991). Genomic (Park et al., 2021), transcriptomic (Wang et al., 2019), and proteomic (Andersen et al., 2023) data consistently revealed the pathways associated with acetate, butyrate, and ethanol production by *En*. *caudatum*. These new insights contribute to the comprehension of alteration in VFA profiles resulting from changes in protozoal populations in response to dietary interventions.

The substantial protozoan biomass and high digestibility of rumen protozoa underscore their importance as a microbial protein source for the host. However, their substantive contribution to the intraruminal recycling of microbial protein concurrently lowers microbial protein supply to the host and reduce dietary N utilization efficiency. Rumen protozoa contain more UFA than bacteria; thus, they can be an important source of beneficial fatty acids. Holotrichids, specifically, may contribute ∼27% to the total rumen lipids (Patel, 2018). Furthermore, rumen protozoa can reduce biohydrogenation of UFA by preying on biohydrogenating bacteria (Yáñez-Ruiz et al., 2007) and storing CLA and vaccenic acid (Devillard et al., 2006). Thus, rumen protozoa can protect UFA from bacterial biohydrogenation. Quantitative appraisals are needed to estimate the beneficial role of individual protozoal groups.

Dairy cows emit CH_4_ during rumen fermentation. A meta-analysis revealed a linear relationship between CH_4_ emissions and protozoa abundance (Guyader et al., 2014). Another meta-analysis focusing on defaunation studies demonstrated that eliminating rumen protozoa could decrease CH_4_ emissions by 11%, accompanied by decreased OM digestibility by 7% (Newbold et al., 2015). Dai et al. (2022) demonstrated that isotrichids might promote CH_4_ production more than entodiniomorphids, suggesting variability in the ability to stimulate CH_4_ emissions among rumen protozoa. The stimulation of CH_4_ by rumen protozoa stems from their ability to produce hydrogen during fermentation within their hydrogenosomes (e.g., species of *Epidinium*, *Dasytricha*, and *Isotricha*) or in their cytosol (e.g., species of *Entodinium*) and to form symbiosis with methanogens (Belanche et al., 2014). Genomic (Park et al., 2021), transcriptomic (Wang et al., 2019), and proteomic (Andersen et al., 2023) data revealed that [FeFe]-hydrogenases are the hydrogen-producing enzymes irrespective of the presence of hydrogenosomes.

Furthermore, rumen protozoa can promote CH_4_ production through ecto- and endo-symbiotic relationships with methanogens. While identifying true symbiotic methanogens continues to encounter challenges, primarily attributable to technical limitations in distinguishing them from engulfed methanogens, recent genomic (Park et al., 2021) and transcriptomic (Wang et al., 2019) studies revealed genes and transcripts involved in symbiosis in *En. caudatum*. As in nonrumen anaerobic protozoa, endosymbiotic methanogens probably reside near hydrogenosomes in rumen protozoa, but direct evidence is lacking. Nevertheless, dietary interventions that reduce rumen protozoa can mitigate CH_4_ emissions (Almeida et al., 2021).

Dairy cows derive 60% to 70% of their metabolizable N as microbial protein synthesized from dietary N. However, only ∼30% of dietary N is retained in milk. A primary factor contributing to this low N efficiency is the intraruminal recycling of microbial protein (Figure 1), which converts a considerable portion of the microbial protein to NH_3_, much of which is excreted as urea. Rumen protozoa constitute a primary driver of this wasteful process (Hartinger et al., 2018). To elaborate, protozoa prey on and digest large numbers of prokaryotes and other smaller microbes in the rumen. Notably, certain rumen protozoa, such as *En. caudatum* and *Epidinium caudatum*, may selectively prey on rumen prokaryotes (Park and Yu, 2018). Using their digestive enzymes (e.g., lysozyme, chitinase, and peptidase), protozoa disintegrate engulfed microbial cells and digest the microbial protein into peptides, free AA, and NH_3_. While some urea is recycled back to the rumen and utilized by bacteria for microbial protein synthesis, a substantial amount is converted to urea and excreted. Thus, rumen protozoa drive intraruminal recycling of microbial protein, reducing the microbial protein supply to animals.

**Figure 1:**
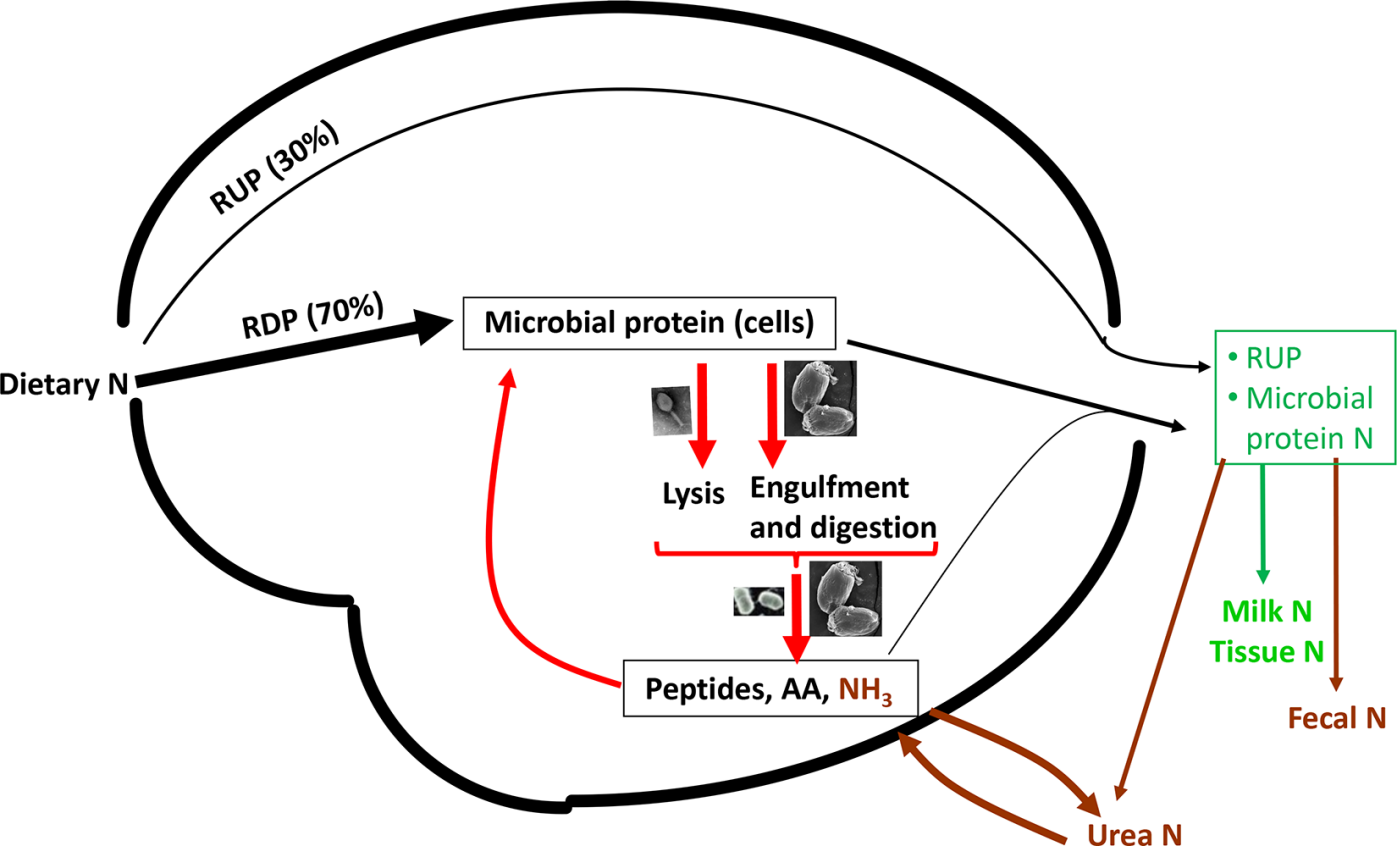
A conceptual model of intraruminal recycling of microbial protein (red arrows). Protozoa engulf and lyse microbial cells from the outside, whereas lytic phages lyse their host cells from the inside. Protozoa and proteolytic (including AA-fermenting) bacteria degrade microbial protein to peptides, AA, and NH_3_. Some of the peptides, AA, and NH_3_ are reused for the synthesis of microbial protein or flow to the small intestines, whereas much NH_3_ is absorbed and converted to urea. Some of the urea diffuse to the rumen.

Rumen protozoa also enhance bacterial proteolysis. On the one hand, microbial protein undegraded or partially degraded by rumen protozoa becomes accessible for bacterial degradation. On the other hand, protozoa extensively lyse in the rumen, with a reported rate of 50% to 80% in steers (Ankrah et al., 1990), making their cellular protein available for proteolysis by bacteria (Park et al., 2019a). While some of the released free AA are used in microbial protein synthesis, a substantial portion is fermented by bacteria, including hyper ammonia-producing bacteria. The contribution of rumen protozoa to intraruminal recycling of microbial protein is illustrated by elevated bacterial abundance, decreased rumen NH_3_ concentration, increased microbial protein flow to the small intestines, improved N efficiency in defaunated animals (Newbold et al., 2015), and a concomitant decrease in NH_3_ concentration when rumen protozoa were inhibited (Park et al., 2019b).

Protozoal predation entails a multifaceted cellular process. Recent omics studies have elucidated that it involves endocytosis, phagosome, lysosome, and cell motility in *En. caudatum* (Wang et al., 2019; Park et al., 2021; Andersen et al., 2023). Entodiniomorphids exhibit higher predatory activity compared to isotrichids. This is evidenced by elevated rumen NH_3_ concentration and a higher abundance of entodiniomorphids in goats relative to cattle (Martin et al., 2021) and upregulated proteins involved in endocytosis in *En. caudatum* in goats (Andersen et al., 2023). Given that rumen protozoa are not indispensable for rumen functions (Newbold et al., 2015), targeted control of specific rumen protozoa, particularly those contributing little to fiber digestion, such as species of *Entodinium*, offers a viable strategy to reduce intraruminal recycling of microbial protein and enhance dietary N efficiency. Recent in vitro studies have demonstrated the feasibility of inhibiting rumen protozoa with specific inhibitors of lysozyme and peptidases (Park et al., 2019a,b) or plants (Ayemele et al., 2021) without impairing feed digestion or fermentation. Integrating genomics, transcriptomics, and proteomics holds promise for identifying specific targets to control rumen protozoa effectively without collateral damage.

Viruses, including those infecting bacteria (bacteriophages), archaea (archaeaphages), fungi (mycophages), and protozoa (protozoan viruses), are abundant in the rumen, reaching 10^7^ to 10^10^ virions/mL of rumen fluid (Lobo and Faciola, 2021). These viruses have 2 distinct lifecycles: the lytic and the lysogenic cycles. Lytic viruses lyse their host cells to release progeny viruses for subsequent rounds of infection. The viral lysis also releases host cellular components, increasing nutrient cycling in the rumen. In the lysogenic cycle, viruses insert their genomes into the host genomes as prophages, leading to lysogeny. Lysogenic viruses can provide their host with new metabolic capabilities, buttressing ecological fitness and potentially facilitating host evolution. Therefore, rumen viruses can profoundly affect the rumen microbiome, its function, and eventually animal productivity.

Early culture-dependent studies documented bacteriophages capable of infecting species or strains of rumen bacteria, including prevalent species of *Prevotella*, *Ruminococcus*, *Streptococcus*, and methanogens (Luo et al., 2001). Analyses of viral metagenomes or bulk metagenomes of the rumen have unveiled a vast diversity of the rumen virome and provided new insights into its potential roles (Anderson et al., 2017). A recent comprehensive analysis of rumen metagenomes derived from ruminant species and geographic regions resulted in the first rumen virome database (**RVD**) containing almost 400,000 species of rumen viruses, with most of these viral species representing new genera or families (Yan et al., 2023). A core rumen virome exists despite variations among animal species, breeds, and geography (Yan et al., 2023). The diverse rumen viruses can infect most lineages of the rumen microbiomes, including 1,051 genera of bacteria, 25 genera of archaea, 13 genera of protozoa (Yan et al., 2023), and fungi (Hitch et al., 2019). Notably, these genera are among those predominant in the core rumen microbiome, including *Prevotella*, *Ruminococcus*, *Fibrobacter*, *Butyrivibrio*, *Megasphaera*, *Streptococcus*, *Clostridium*, *Methanobrevibacter*, and *Entodinium* (Yan et al., 2023). Thus, the rumen virome potentially affects the diversity, composition, metabolism, and functions of the rumen ecosystem.

Viruses can exert a top-down influence on the abundance of their host species by lysing them. By killing the winner, lytic phages predominantly “prey on” fast-growing microbes, thereby preventing slower-growing microbes from being completely excluded and helping maintain the diversity and dynamic balance among different microbes (Chen et al., 2021; Marantos et al., 2022). Although the “kill the winner” model comes from studies in other ecosystems, it shall apply to the rumen ecosystem. However, a recent study revealed a lower viral-host ratio in cell-dense habitats like the gut, compared with the ocean (López-García et al., 2023). A different virus-host interaction dynamic, “piggyback the winner” (Knowles et al., 2016), might be more common within the rumen. Interestingly, prophages are identified in nearly half of the thousands of examined rumen microbial genomes, with extreme cases containing up to 8 prophages within a single genome (M. Yan and Z. Yu, unpublished data), underscoring the significance of lysogeny within the rumen ecosystem. Due to differences in cell density and microbial turnover rate among epimural, fiber-adherent, and planktonic communities, the dynamics of virus-microbe interactions may vary within these microbial communities, which justifies the need for spatial analysis. This variation highlights the importance of considering spatial factors when studying these interactions.

Viruses exhibit variability in the species they infect, ranging from specific strains within the same species to a diverse array of species spanning multiple phyla (Yan et al., 2023). Rumen viruses infect degraders of fiber (e.g., species of *Prevotella*, *Ruminococcus*, *Fibrobacter*), starch (e.g., *Streptococcus*, *Ruminobacter*), protein (*Prevotella*, *Butyrivibrio*), sugar fermenters (e.g., *Selenomonas*), methanogens (e.g., *Methanobrevibacter*), and protozoa (e.g., *Entodinium*). Furthermore, many rumen microbes adhere to solid feed particles, forming a biofilm especially important for fiber digestion (De Mulder et al., 2017). Viruses can facilitate biofilm formation by releasing extracellular DNA and other means (Rice et al., 2009), but they can also disrupt biofilm by lysing their hosts and depolymerizing exopolysaccharides (Hughes et al., 1998). While not yet experimentally validated, rumen viruses probably also affect the dynamics of the biofilm on solid feed particles. Hence, the rumen virome probably affects key rumen functions by regulating the populations of predominant rumen microbes, including key guilds and those forming biofilm.

Viruses play a crucial role in nutrient recycling in aquatic ecosystems (Roux et al., 2016). While not yet experimentally verified, it is likely that rumen viruses also mediate the cycling of nutrients in the rumen, including carbohydrates, lipids, and protein. Of particular importance in ruminant nutrition is their influence on microbial protein metabolism. By lysing their hosts, rumen viruses increase the availability of microbial protein for bacterial proteolysis, thereby contributing to the intraruminal recycling of microbial protein (Figure 1). Additionally, viral lysis reduces the population of the hosts, thus reducing microbial protein synthesis. Furthermore, viral lysis of key guilds, such as fiber and starch degraders, may unbalance energy and N metabolism, further reducing microbial protein synthesis. However, the presence of protozoal viruses adds complexity to the influence of rumen virome on dietary N efficiency by lysing protozoa.

Examining the RVD, Yan et al. (2023) found numerous AMG representing 41 distinct categories. These “unessential” AMG can repurpose, manipulate, or augment host metabolism (Warwick-Dugdale et al., 2019). For example, AMG can encode enzymes involved in feed digestion (e.g., GH) and protein synthesis (e.g., asparagine synthase), as well as nucleotide metabolism, signaling, transportation, and counter-defense (Yan et al., 2023). Viral AMG thus potentially affect the rumen microbiome and its functions in a bottom-up manner. Additionally, viruses can facilitate host evolution, speciation, and ecological fitness through generalized, specialized, or lateral transductions. Future research is warranted to demonstrate the potential and assess the magnitude of the impact of the virome on the microbiome within the rumen ecosystem and animal productivity.

Rumen protozoa and viruses, acting as predators within the rumen microbiome, remain less understood compared with bacteria and methanogens, primarily due to challenges in their definitive characterization through culturing. Recent omics studies have greatly advanced our understanding of these rumen microbes, providing new insights into their crucial roles in shaping the rumen microbiome and its functions. In the context of dairy production, exploring certain rumen viruses may offer novel strategies to control undesirable rumen microbes, including methanogens, protozoa, *Streptococcus* species, and hyper ammonia-producing bacteria. Notably, one study has demonstrated that a viral enzyme (PeiR, an endopeptidase) from one *M. ruminantium* phage inhibited rumen methanogens, resulting in a remarkable up to 97% reduction in CH_4_ production in vitro (Altermann et al., 2018). Lytic phages infecting *Streptococcus* species could be explored to inhibit this bacterial species and reduce the risk of rumen acidosis in cows fed high-concentrate diets. This potential has been demonstrated by the inhibition of *S. equinus* with endolysin LyJH307 (Kim et al., 2021). Similarly, the phages infecting rumen protozoa, particularly those highly bacterivorous (e.g., species of *Entodinium*) and those infecting hyper ammonia-producing bacteria, hold the potential to reduce intraruminal recycling of microbial protein and deamination, thereby enhancing dietary N efficiency. Research in this area can contribute to a more sustainable dairy industry by helping reduce rumen acidosis, CH_4_ emissions, and N excretion.
